# Anemia in young women: determinants and artificial intelligence-based management approaches

**DOI:** 10.3389/frai.2026.1747611

**Published:** 2026-02-19

**Authors:** Guttapalam Sireesha, D. Madhavi, A. M. Beulah, M. Niharika, C. Pradeepthi

**Affiliations:** Sri Padmavati Mahila Visvavidyalayam, Tirupati, India

**Keywords:** anemia, artificial intelligence, hemoglobin, management, young women

## Abstract

Anemia is a serious global public health problem, worldwide majority of the young women are suffering with this anemia. Anemia condition is characterized by the deficiency iron, folic acid and other nutrients. Not only nutritional deficiencies, some other factors like environmental, genetic, physiological, nutritional, urbanization and socioeconomic factors influencing the anemia condition. Anemia is highly prevalent and has significant health and economic consequences efforts to decrease its prevalence in this young women group have been surprisingly slow. An Artificial Intelligence helps to shift in addressing the anemic problem. This review focusing on the multifactorial causes of anemia in young women and also AI- based interventions for screening, risk assessment, personalized nutritional counseling, treatment, management and also public health monitoring. AI facilitates greater accessibility, and personalized treatment, its responsible application requires careful consideration of algorithmic biases, data quality, ethical, and seamless integration with current healthcare systems. AI has the potential to revolutionize anemia management and promote equitable responsible and effective health outcomes for young women worldwide.

## 1 Introduction

Anemia is characterized by low concentration of the hemoglobin or lower number of blood cells than the normal, due to this, the body’s ability to transport oxygen to tissues and organs is significantly reduced (Dwomoh et al., 2025). The condition remains a common health issue that has an specifically negative effect on vulnerable groups like young children, adolescents who are menstruating, pregnant women, and postpartum women (WHO, 2024) Anemia is more than just a medical diagnosis. Anemia is s serious public health issue which has a significant effect on the both human health and nutritional development.

### 1.1 Global burden of anemia in young adult women

Anemia affects people of all ages and social backgrounds and it is a major public health problem worldwide. Anemia is most prevalent in developing countries (Behera et al., 2024). Majority of the women and children are disproportionately affected by anemic condition worldwide, making them the most vulnerable groups. 2019 statistics showing that an approximately 30% of women aged 15 to 49 years were anemic. In 2023 there is no improvement in the anemic percentage of non-pregnant and pregnant women 15–49 years (WHO, 2025). It clearly indicates that approximately half a billion women of reproductive age are anemic worldwide (Merid et al., 2023). But the impact of anemia is different, the burden is greatest in low and lower- middle -income countries (LMICs), where the condition is more common in rural areas, among low- income households, and among populations with less access to formal education (WHO, 2025). This is a significant socioeconomic and developmental barrier, as evidenced by its persistently high prevalence, particularly in LMICs and among vulnerable population. It slows the development of human capital and starts a cycle of sickness and poverty. When women are overloaded with work, caring for their families, or participating in education and decision- making, the negative impact is generational, reducing productivity, educational attainment, and cognitive ability (Merid et al., 2023). There is an urgent need to reassess and potentially disrupt strategies, as current global efforts are not enough to achieve the 2030 targrt of a 50% reduction in the prevalence of anemia (WHO, 2024).

### 1.2 Overview of AI’s potential in healthcare

Artificial intelligence (AI) is transforming healthcare rapidly, offering revolutionary opportunities for disease prediction, early diagnosis, and preventative treatment. By identifying complex patterns in large medical date sets that humans cannot see or predict, AI dramatically improves diagnostic accuracy through advanced machine learning (ML) algorithms, deep learning networks (DL), and advanced big date analytic (Ahmed et al., 2020). Convolutional neural networks (CNNs), Recurrent Neutral Networks (RNNs) and Support Vector Machines (SVMs) are examples of AI models that have established significant success in improving early diagnosis rates for numerous diseases, including diabetes, heart disease, and certain types of cancer (Khalifa and Albadawy, 2024).

Beyond diagnosis AI has enormous potential to improve patient care efficiency, streamline treatment planning, and reduce overall healthcare costs (One Sec Magazine, 2025). A new way to overcome the inherent shortcomings of tradition anemia diagnosis and treatment is AI’s advanced data analysis and patter recognition capabilities. Traditional approaches to disease diagnosis often rely on subjective assessment and late onset of symptoms, leading to delays in treatment and high mortality rates (Khalifa and Albadawy, 2024). Furthermore, traditional anemia diagnosis can be invasive, time- consuming, and inconvenient for patients (Sehar et al., 2025). However, the ability of AI to process and understand large and complex data sets creates opportunities for earlier, more accurate, and more personalized interventions, especially in settings with limited access to traditional diagnostic infrastructure. This stark contrast demonstrates AI’s potential to address and even eliminate the current errors and mistakes in the treatment of anemia (Khalifa and Albadawy, 2024).

### 1.3 Purpose and scope of the review

In the context of present study “young adult women” are basically considered as those aged 18 to 26 years, although some of them, due to their better development, fall into the age group of 16 to 30 years (NAHIC, 2025). People usually become more independent, develop lifelong health habits, and take on adult responsibilities during this age range which is acknowledged as a crucial transitional period (Science Focus, 2025). According to society, 18 is the “age of majority” and 26 is frequently mentioned as the age at which many people have finished the normal adult transitions and are becoming established as adults (NAHIC, 2025).

Conditions like mental disorders and sexual health problems, which include the highest rate of unplanned pregnancy among women aged 18 to 24. Often start in young adulthood Significant socioeconomic vulnerabilities also define group; for example, 74% of 18 to 25 years- olds make less than $25,000 annually, which can have a negative impact on their long—term health (Science Focus, 2025). Together, these elements highlight the significance of targeted health interventions for young adult women, a demographic that is both physiologically vulnerable to anemia and frequently encounters structural obstacles to receiving healthcare and achieving the best possible health outcomes (Kumar et al., 2022).

Present review aims to provide an overview of the current knowledge on anemia in young women. Information is particularly relevant for women aged 15–49 years represent the age group with the highest risk of anemia. To fully understand the potential of artificial intelligence to promote health equity, this review article aims to assess the effectiveness of current applications of AI, analyze the main limitations and ethical issues associated with their use, and suggest future directions for research and policy action.

## 2 Anemia in young adult women

### 2.1 Definition, diagnostic criteria, and classification

A decrease in the red blood cells number or a lower-than-normal concentration of hemoglobin (Hb) is the two main characteristics of anemia, several physiological consequences result from this deficiency, affecting the blood’s ability to effectively oxygen to the body’s cells and organs (WHO, 2025).

The World Health Orgaization (WHO) states that certain hemoglobin thresholds are used to diagnose anemia. Anemia is defined as a hemoglobin level below 12.0 g/dL in non-pregnant women and below 11.0 g/di in pregnant women (Behera et al., 2024). Table 1 shows the WHO/CDC classification and diagnostic criteria for anemia in women. Different are used to further classify the severity of anemia:

**Table 1:** WHO/CDC anemia classification and diagnostic criteria for women.

Category	Severity	Hemoglobin (Hb) Cut-off (g/dL)
Non-pregnant women	Any Anemia	< 12.0
	Mild Anemia	11.0–11.9
	Moderate Anemia	8.0–10.9
	Severe Anemia	< 8.0
Pregnant women	Any Anemia	< 11.0
	Mild Anemia	10.0–10.9
	Moderate Anemia	7.0–9.9
	Severe Anemia	< 7.0

Normal Hb distribution varies with sex, ethnicity, physiological status, and altitude. Higher altitude requires upward adjustment of Hb cutoffs.

Mild anemia by an Hb level of 11.0 to 11.9 g/dL in non-pregnant women and 10.0 to 10.9 g/dL in pregnant women. Moderate anemia by hemoglobin levels of 8.0 to 10.9 g/ dL in non-pregnant women and 7.0 to 9.9 g/dL in pregnant women. Severe anemia by hemoglobin levels fall below 8.0 g/ dL in non-pregnant women and below 7.0 g/dL in pregnant women (Children's National, 2025).

It is important to understand that a person’s sex, ethnicity, and physiological state can all have a substantial impact on their typical hemoglobin distribution (Behera et al., 2024). For instance, because the oxygen partial pressure is lower at higher elevations residents need to have their Hb cutoffs adjusted upward (Children's National, 2025). A thorough evaluation that takes into account hematologic markers, knowledge of the underlying pathogenic mechanisms, and a thorough patient history is necessary for the diagnosis of anemia, which is frequently multifactorial (Behera et al., 2024). Because a complete blood count (CBC) may not be able to identify iron deficiency in its early stages, a second blood test to quantify ferritin protein is often necessary for illnesses such as iron insufficiency (Channar et al., 2023). Given the complex nature of anemia and the variation in Hb cutoffs depending on altitude, ethnicity, and phycological Status (pregnancy), comprehensive and context- specific diagnostic methods are essential. The limits of generic cutoffs or subjective clinical judgments may be overcome by AI, which has the potential to do this by combining numerous data points beyond simple Hb levels and providing a more relevent, precise, and context—aware diagnostic assessment (Pawuś et al., 2024).

### 2.2 Global and regional prevalence

As previously mentioned, 30% of women worldwide between the ages of 15 and 49 years suffered from anemia in 2019 (WHO, 2025). It was slightly more common by 2023, affecting 35.5 and 30.7% pregnant and non-pregnant women in 15 to 49 years age range (WHO, 2024).

Low- and middle—income countries (LMICs), particularly those in the WHO regions of Africa and South- East Asia, bear the greatest burden of potentially severe anemia. An estimated 106 million women in Africa and 244 million in South—East Asis are anemic, the highest percentage of women living in these regions alone (WHO, 2025).

Despite of continued international efforts and initiatives, the prevalence of anemia remains high. The global target of reducing anemia by 50% 2030 is far from being achieved, and there are significant barriers to achieving the Global Nutrition Target (GNT), which calls for a 50% reduction in anemia among women of reproductive age (WHO, 2024). Theis high incidence, which continues over time, highlights the limitations of current interventions and the urgent need for creative, scalable solution. The high prevalence of chronic anemia reflects the fact that current public health strategies, although valuable are not sufficient to prevent this disease. AI technologies and its implementation and usage will improve the global health as revolutionary way.

### 2.3 Impact of anemia on health, social and economic

Anemia can cause a number of symptoms that severely reduce a person’s productivity and overall well- being, Extreme fatigue, decreased physical performance, shortness of breath, dizziness or fainting, cold hands and feet, headaches, and flushing of the skin or mucous membranes are common health effects. In more extreme situations, symptoms, may worsen and include elevated bruising, fast heartbeat, and quick breathing (WHO, 2025). Neurological symptoms, such as numbness, muscle weakness, psychological problems (from mild depression to confusion and dementia), balance and coordination issues, and pins and needles, are also noted for certain types of anemia, such as those brought on by vitamin B12 or folate deficiency (Franciscan Health, 2025). Anemia effects are not limited to the individual: in children severe anemia can hinder cognitive and motor development and in pregnant women increase the risk of birth difficulties, which can result in low-birth-weight newborns, delayed development, and compromised immune systems in their offspring (WHO, 2025).

A harmful intergenerational cycle is produced by the severe health effects of anemia, especially on physical and cognitive abilities. Particularly iron deficiency in women are greatly hindered from realizing their full potential by anemia, which causes persistent fatigue, compromised immunity, and diminished cognitive function (One Sec Magazine, 2025). Women who are too worn out to work, take care of their families, or engage in education and decision—making have a direct impact on the welfare of their households and the larger development of their communities’ women are the main contributors to productivity in many economies, especially those that depend on agriculture and caregiving. Communities suffer as a result of general productivity declines brought on by iron deficiency (One Sec Magazine, 2025). Especially, anemia in young women impairs cognitive function, labor productivity, educational attainment, and mental wellness (Merid et al., 2023). Theis illustrates a definite cause—and—effect link in which a health condition has a direct impact on a person’s potential, the well- being of their family, and the growth of the community as a whole, resulting in a vicious cycle of poverty and bad health outcomes that persists for generations. Therefore, treating anemia is not just a medical procedure but also a vital investment in the advancement of society and individuals.

## 3 Key determinants of anemia in young adult women

**Figure 1:**
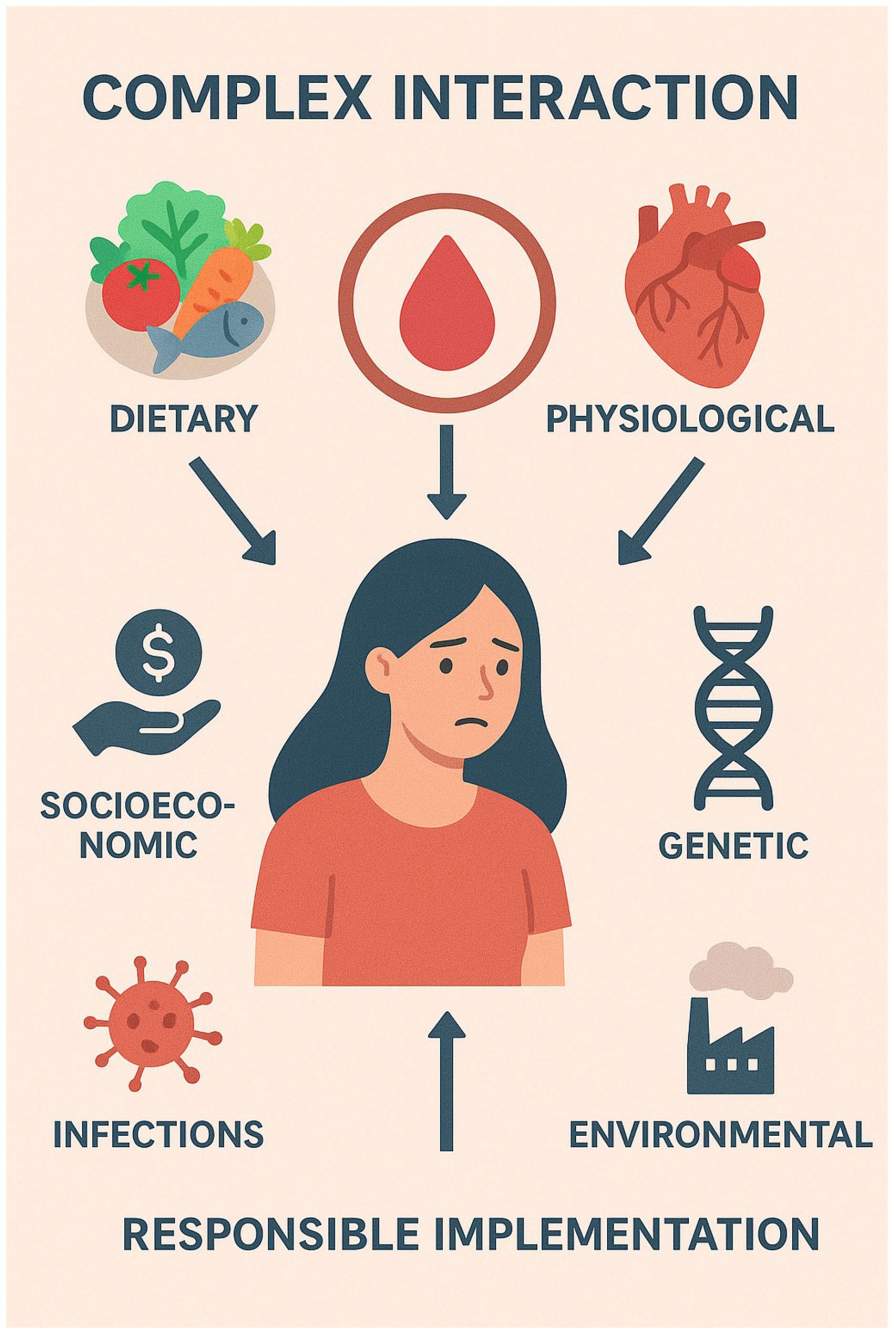
Key determinants of anemia in young adult women.

A Complex interaction of dietary, physiological, socioeconomic, genetic, infectious, and environmental variables can lead to anemia in young adult women (Figure 1). It is important to comprehend the effecting factors in order to create prevention plans that work to treat anemia.

### 3.1 Nutritional deficiencies

Iron deficiency anemia most prevalent in worldwide (WHO, 2025). The pathogenesis, of iron functions in hemoglobin synthesis and erythrocyte formation, deficits in other essential micronutrients; including vitamin A, Folate, vitamin B12, and riboflavin, also play important roles (WHO, 2025). Number of factors are causing for low iron levels in young women. Because the monthly blood loss severely, depletes iron stores, heavy menstrual bleeding is recognized as a major physiological risk factor, especially in younger women (Kario and Kurniawan, 2024). In the pregnancy time significant raise of iron was observed, due to the increase of the red blood cell volume in younger fetus (Georgieff, 2020). Anemia and a higher chance of unfavorable delivery outcomes, such as low birth weight babies, result when this increased demand frequently exceeds nutritional intake (Merid et al., 2023). Moreover, one common dietary reason, vegetarians and vegans are more likely to get iron deficiency (Kario and Kurniawan, 2024) which comes from plant sources including spinach, beans, legumes, nuts and fortified cereals, because these are less accessible non-heme iron sources. Where as in animal sources like meat and poultry contains heme iron which is more easily absorbed by the body (Kario and Kurniawan, 2024).

An effective dietary interventions are essential. For prevention and management of nutritional anemia. This involves eating a healthy balanced, diet that includes foods which are high in iron folate, vitamin B12, and Dietary supplements may recommend (WHO, 2025). The difference of heme and non- heme iron absorption is the primary cause for anemia and the need for other micronutrients underscore the challenge of successfully implementing dietary therapy (Soni et al., 2025). AI can play a main role in providing personalized individual dietary recommendations that go beyond general recommendations to address specific deficiencies related to each individual’s it maximize the nutrient absorption based on individual needs. AI can optimize nutrient intake and address specific deficiencies than tradition general nutritional advice by analyzing an individual’s unique dietary habits, genetic predispositions, metabolism, and other heath data to provide personalized recommendations that take into account (NHS Inform, 2025).

### 3.2 Socioeconomic factors

The anemia prevalence is significantly influenced by socioeconomic status. It includes Low income, limited access to health services, living in rural areas, and educational illetarcy are important factors that strongly contribute to an increased risk of anemia (DHS Program, 2025). The risk of anemia is increased by low educational attainment, with a poor understanding of the importance of nutrition, especially during pregnancy (Davidson et al., 2022). The prevalence of anemia is also driven by the lack of adequate social support unstable employment or low household income, which further increasing the risk of anemia (Davidson et al., 2022).

Directly linked with access to health foods and high- quality health care, especially basic prenatal care, low socioeconomic status of individuals and families are (Sehar et al., 2025).

These socioeconomic conditions create a complex web of disadvantage. Understanding this broader problem is crucial, as it suggests that interventions must be multifaceted and address not only medical, but also socioeconomic factors (Davidson et al., 2022). This ins’ t merely a collection of isolated factors—it is a systemic issue where one problem, like poor health, can trigger a chain reaction, limiting access to essential needs such as nutritious food and a health way of life. This leads to a vicious cycle of vulnerability to anemia.

### 3.3 Physiological and health factors

Young women is a significantly higher risk of anemia due to unique biological vulnerabilities, which are further face a compounded by chronic illnesses and genetic predispositions.

#### 3.3.1 Menstruation

Excessive or menorrhagia menstrual bleeding is a major physiological risk factor for iron deficiency anemia in young women, due to the significant monthly repeated blood loss can deplete iron stores over time and it associated with their reproductive biology (Munro, 2023; Lee, 2020).

Monthly heavy blood loss during menstruation depletes the body’s iron stores, and if this deficiency is not replaced through diet or supplementation, anemia can develop (Georgieff, 2020). Anemia is common and often undiagnosed condition, which may lead to the prevalence of iron deficiency among women (Pan et al., 2025). The severity of the menstrual bleeding in women need to alter pads or tampons more than once per 2 h or passing substantial blood clots at increased risk (DeLoughery et al., 2024).

#### 3.3.2 Pregnancy

This condition raises the need of iron dramatically as the volume of red blood cells in the mother’s body increases to sustain her own body and the growing fetus. Throughout this period, insufficient intake of iron can cause anemia, which increases risks of negative birth outcomes, such as low birth weight babies and other complications (Merid et al., 2023). Young women are different from older reproductive- age women in nutritional needs with the risk of anemia raises during adolescence with the addition of menstruation and pregnancy (Merid et al., 2023).

#### 3.3.3 Chronic diseases

One third percentage of anemic due to chronic disease. Chronic inflammation, acquired resistance of bone marrow erythroid progenitors to erythropoietin can underlie unexplained anemia (Behera et al., 2024).

#### 3.3.4 Genetic conditions

An individual’s vulnerability to anemia can be greatly influenced by genetic predisposition, even if nutritional and physiological factors are equally common. Certain forms of anemia are inherited directly, which means that families pass them on (Munro, 2023).

Iron—refractory iron deficiency anemia (IRIDA) is a prominent hereditary type. This uncommon disorder is caused by mutations in the TMPRSS6 gene, which controls the body’s iron levels (Sharma et al., 2024). The significance of genetic testing for differential diagnosis is highlighted by the fact that people with IRIDA may exhibit symptoms that bare comparable to those of other types of anemia yet may not improve with traditional iron supplementation. Due to the autosomal recessive inheritance pattern pf IRIDA, a child cannot be impacted until both parents have the recessive trait, even if neither parent exhibits any symptoms (WHO, 2025).

##### 3.3.4.1 Other inherited conditions that lead to anemia include

###### 3.3.4.1.1 Sickle cell disease (SCD)

This genetic disease two aberrant copies of the hemoglobin—producing B-globin gene are inherited (Wikipedia Contributors, 2025). An estimated 7.7 million people are thought to be affected globally, with Sub-Saharan Africa accounting for nearly 80% of cases (Wikipedia Contributors, 2025). Consequences of sickle cell disease (SCD) is chronic anemia, organ damage, permanent disability low quality of life, and early death (Da Silva Brito et al., 2022).

###### 3.3.4.1.2 Sickie cell trait (SCT)

SCT Patients are typically asymptomatic, have a mortality rate and quality of life comparable to the general population, and inherit one faulty copy of the B-globin gene (Behera et al., 2024). With a 9% prevalence rate among African Americans, SCT is more common in those of African heritage and those from tropical and subtropical areas where malaria is endemic (Da Silva Brito et al., 2022).

###### 3.3.4.1.3 Thalassemia

Alternative genetic medicines anemia transformed by beta- thalassemia (BTT) (Science Focus, 2025). Artificial intelligence (AI) model has shown promise in anticipation of high difference in the form of anemia (Sehar et al., 2025).

###### 3.3.4.1.4 Infection

Anemia is primarily caused by infections, especially parasitic infections such as malaria, especially in resource—limited settings (WHO, 2025).

And in a special vulnerable biology, genetics predisposed to affective chronicity: the adult woman needs specialized screening because she needs preventive screening that is tailored to preventive conditions or specific physiology. Biologically complex individuals are susceptible to disease and require community—based care strategies. Table 2 enlists the main factors of care that contribute to anemia from women adults.

**Table 2:** Key determinants of anemia in young adult women.

Category	Specific determinants	Brief impact/mechanism
Nutritional deficiencies	Iron deficiency	Effects hemoglobin synthesis.
	Folate deficiency	Impaired red blood cell production.
	Vitamin B12 deficiency	Impaired red blood cell production and neurological issues.
	Vitamin A deficiency, Riboflavin deficiency	Effect in hemoglobin synthesis and erythrocyte production.
Socioeconomic factors	Low income	Limits access to nutritious food and quality healthcare.
	Low education	Limited knowledge about nutrition and health.
	Limited healthcare access	Hinders early detection and intervention.
	Residing in remote/rural areas	Reduced access to healthcare and nutritious food.
	Lack of social support	Contributes to increased anemia incidence.
Physiological factors	Heavy menstruation	Significant monthly blood loss depletes iron stores.
	Pregnancy	Increased demand for iron and nutrients for maternal and fetal health.
Genetic factors	Sickle Cell Disease (SCD)	Inherited abnormal hemoglobin, chronic anemia, organ damage.
	Sickle Cell Trait (SCT)	Carrier state, generally asymptomatic but important for genetic counseling.
	Thalassemia	Inherited blood disorder affecting hemoglobin production.
Other health factors	Chronic diseases	Anemia of chronic disease, inflammation, erythropoietin resistance.
	Infections (e.g., Malaria)	Increased inflammation, nutrient loss, red blood cell destruction.

### 3.4 Environmental factors

Environmental factors and socioeconomic conditions, they are directly linked to influence the incidence of anemia. These include elements of the physical environment and broader social factors that influence health.

Access to clean water, hygiene and sanitation (WASH) is essential. Particularly among young women insufficient supplies of safe drinking water and inadequate sanitation are associated with a higher risk of anemia (Merid et al., 2023). Inadequate WASH (Water, Sanitation, and Hygiene) facilities contribute to the spread of infection diseases—including parasitic infection like schistosomiasis and soil—transmitted helminths, as well as diarrheal illnesses—which can cause chronic blood loss, inflammation, and nutrient malabsorption, ultimately increasing the risk of anemia. Prevention of anemia requires immediate attention on addressing these environmental factors in addition to poverty and illiteracy (WHO, 2025).

The impact of environmental inequalities is reflected in the global prevalence of anemia, which is more common in rural areas and among low-income families (WHO, 2024). The risk of anemia can also be influenced by the general environment, including exposure to harmful chemicals and living can be affected by a wide range of environmental influences (Munro, 2023).

## 4 The evolving role of AI in Anemia management

The integration of artificial intelligence (AI) into chronic disease management holds significant potential to enhance patient care efficiency, optimize treatment strategies, and drive the development of innovative healthcare solutions (Pan et al., 2025). This segment reviews various application areas and demonstrates the effectiveness of artificial intelligence (AI) in the treatment of anemia.

### 4.1 AI applications in disease prediction and early diagnosis

Artificial intelligence plays a transformative role in improving diagnostic accuracy by identifying complex patterns using large medical datasets (Khalifa and Albadawy, 2024). Several machine learning (ML) and deep learning (DL) algorithms, including convolutional neural networks (CNN), support vector machines (SVM), naïve Bayesian systems, XGBoost, Random Forest, and Extreme Learning Machines (ELM), are being developed and applied for early diagnosis and classification of various types of anemia (Khalifa and Albadawy, 2024).

Through various non-invasive diagnostic methods a significant progress is made. These methods have been shown to be effective, cheaper, and faster than traditional invasive blood tests they are conjunctival, palm or nail image analysis (Subramanian et al., 2020). In particular, AI- powered smartphone applications that can estimate hemoglobin (Hb) levels via “nail selfies” with high accuracy show a mean absolute error of = 0.7 g/dL, improving to = 0.50 g/Dl, for Hb levels above 10 g/Dl (Mannino et al., 2025). These smart apps enable convenient self-monitoring and have been widely used in the real world with over 1.4 million tests prevalence by over 200,000 users, facilitating the first county—level mapping of anemia prevalence in the United States (Mannino et al., 2025). The use of personalized apps for patients with chronic anemia has further improved diagnostic accuracy by nearly 50% (Chapman University, 2025).

Due to their high accuracy non-invasive nature, AI—driven diagnostic tools, particularly smartphone—based solutions, have proven to be effective and affordable screening methods. Traditional diagnostic infrastructure is limited and expensive than the detection through AI, and also this is less expensive, shorter in duration, and reliable for early detection (Subramanian et al., 2020). The high accuracy of these non-invasive methods has been consistently reported, with CNN achieving 90.27% accuracy and ELM 99.21% accuracy for anemia detection, further supporting their practical utility and significant potential for broad public health impact (Subramanian et al., 2020).

Furthermore, AI models can analyze complete blood counts (CBCs) to diagnose and classify specific types of anemia, such as iron deficiency anemia (IDA), beta- thalassemia trait (BTT), and hemoglobin E (HbE), with ELM models achieving up to 99.21% accuracy (Sehar et al., 2025). Several high-throughput machine learning models that can not only detect the presence of anemia but also classify specific types demonstrate that AI can significantly improve diagnostic accuracy. This goes beyond simple detection and resource allocation. Integrating ontological knowledge with machine learning models has also been suggested to further improve classification results and increase the interpretability of AI decisions (Sehar et al., 2025).

### 4.2 AI for personalized nutrition and dietary recommendation

Artificial intelligence and machine learning (AI) can be used to provide personalized nutritional advice and develop advanced strategies. These systems utilize personalized health information, including eating habits and even genetic data, to inform care and treatment decisions (NHS Inform, 2025). AI algorithms analyze large amounts of data to identify very specific nutritional deficiencies and then recommend personalized nutritional programs to address these imbalances (NHS Inform, 2025).

#### 4.2.1 AI–powered dietary assessments and meal planning

For precise analysis of a person’s eating habits and to identify nutritional deficiencies to improve one’s health, AI can be used. Using computer vision, the food images are analyzed for its consumption patterns by calculating the nutritional value by the help of AI powered devices (Gami et al., 2024). They can also integrate data from wearable devices and biometric sensors-such as continuous glucose monitors -to track indicators like blood sugar levels, providing a comprehensive view of an individual’s metabolic response to food (DeLoughery et al., 2024). This objective, real-time data collection is more accurate than traditional self - report methods, which are prone to recall errors (Prajwal et al., 2023).

AI can be used to for personalized and customized meal plans and recipe suggestion based on an individual’s age, gender, health need, activity level, and food preferences. For example, apps like Eat love generate tailored meal recommendation that support healthy eating habits and even assist users with creating shopping lists (Phalle and Gokhale, 2025). This degree of personalization can lead to restrictions which fail to consider individuals differences in metabolism, gut microbiota, genetic factors, and psychosocial contexts (WHO, 2025).

These systems contain multi—source health data which includes medical records, insights from wearable health monitors, and detailed food diaries to construct a comprehensive “health graph.” This multi-modal approach provides a holistic view of a person’s health and nutritional state which leads to more accuracy and thorough recommendation (NHS Inform, 2025). As key feature must be the ability for real time monitoring and dynamic adjustment of dietary recommendations. Whenever users acquire new health information (e.g., blood test results) or have any changes in dietary modification leads their health graphs are instantly updated, thus the AI algorithms provides flexibility, evidence-based suggestions to continuously optimize outcomes (NHS Inform, 2025).

#### 4.2.2 Predictive nutrition and health forecasts

In consideration of historical data on dietary plans and health markers AI alerts the user about potential health issues caused by dietary choices. By continuously monitoring nutrient uptake, AI can notify the users about possible deficiencies such as iron, vitamin A, folate, or B12 even before symptoms arise. This optimism allows adjustments to the dietary plans to avoid the exacerbation of anemia (Phalle and Gokhale, 2025).

AI-generated dietary interventions have shown promise in outperforming traditional approaches, leading to improved health outcomes. Recent studies suggest that AI-driven personalized nutrition technologies can positively influence various health indications, including increasing ferritin levels—an important marker of the body’s iron stores (WHO, 2025). This suggests that AI can effectively guide individuals toward diet that optimizes nutrient absorption and address the specific deficiencies relevant to anemia. The ability of AI to continuously learn and adapt based on treatment outcomes and patient responses in real-time allows for the refinement and optimization of nutritional plans by fostering a more patient—centered approach to care (Ohara et al., 2021).

AI-based personalized nutrition tailors’ general dietary guidelines to individual biological and lifestyle factors, offering a precise approach to managing nutritional anemia. Studies have shown that AI—generated intervention can outperform traditional methods, with six out of nine comparative studies reporting significant improvements in glycemic control, metabolic health, and psychological well-deign (WHO, 2025).

### 4.3 AI–based anemia management systems

The integration of artificial intelligence into chronic disease management has creates opportunities to improve the efficiency of patient care and patient care and optimize various treatment strategies (Pan et al., 2025). Specific AI-driven decision support systems, such as the Anemia Control Model (ACM), have been developed to help physician’s select personalized anemia treatments for patients (Gandjour et al., 2025).

#### 4.3.1 Optimized treatment regimens

AI models can analyze the patient data include hemoglobin levels, mean corpuscular volume (MCV), ferritin, and transferrin saturation (TSAT) along with their tends and the record of medication dosages. This enables AI to predict responses to treatments such as erythropoiesis—stimulating agents (ESAs) and iron supplements (ISs) and to recommend optimal dosages foe improved outcomes (Koo et al., 2024).

An international prospective study in hemodialysis patients demonstrated the effectiveness of the Anemia Control Model (ACM), showing reduced darbepoetin use, improved target hemoglobin levels (up to 83.2% with ACM implementation), decreased hemoglobin variability, and a significantly lower risk of hospitalization (Gandjour et al., 2025). These results suggest that artificial intelligence can improve the use of big data to achieve personalized or precision medicine in anemia management (Subramanian et al., 2020).

AI helps is getting positive outcomes through personalized medicine by analyzing factors such as genetic makeup, lifestyle choices, and environmental factors to create targeted interventions (Vij, 2024). This includes predicting the disease progression, which is crucial for conditions such as chronic anemia to identifying optimal treatment options (de Graaf et al., 2025).

#### 4.3.2 Remote monitoring and patient engagement

AI-enabled wearable technologies and mobile health applications which facilitates real—time patient monitoring allowing for continuous tracking of health parameters relevant to detect anemia such as heart heat, O_2_, sleep patterns, and physical activities (Ohara et al., 2021). AI-based data can provides early detection and warnings of potential complications and ensure timely intervention for at-risk patients by reducing the need for frequent clinic visits (Dasl and Gupta, 2024).

AI enabled tools play a major role for enhancing patient continuous monitoring engagement and compliance of data by providing immediate, 24/7 support, personalized feedback, and reminders. Chatbots and virtual health assistants, employing natural language processing (NLP) that can help patients book appointments, obtain health information, and receive remote care (Gifari et al., 2021). This kind of proactive support empowers patients to take an active role in their persona; care through tailored health plans and helps in building new healthy lifestyle habits improving their quality of life (Mohamed et al., 2024).

Early case studies and observational data demonstrated that AI enabled data interventions can lead to measurable improvements in the clinical outcomes. For patients with chronic anemia, personalized use of smart apps has improved diagnostic by nearly 50% leads to safer and easier home-based treatment management (Chapman University, 2025). This data includes a better reminders in hemoglobin control, reduced usage of medication, and it may lead to decreased hospitalization rates; suggesting a patient health management and patient health management and tangible positive impact on overall quality of life. These are examples illustrate that how the AI, health care management systems are designed to offer a paradigm shift in anemia treatment by providing data - driven, personalized therapeutic guiding to more stable patient outcomes, reduced usage of medicines and potentially lower healthcare costs, particularly for chronic conditions which requires continuous treatment and management.

### 4.4 AI in public health management and epidemiology

AI offers unparalleled opportunities to improve public health outcomes by identifying risk factors detecting patterns and predicting outbreaks to processing massive amounts of data (Paramasivan, 2023). This kind of approach helps in risk assessment, particularly valuable for managing and preventing anemia at a population level.

#### 4.4.1 Surveillance and outbreak prediction

AI–powered predictive analytics can analyze large datasets to forecast trends in health conditions, including anemia prevalence, and identify high-risk populations or geographic regions (Paramasivan, 2023). Hence, AI can be used to map hemoglobin levels geographically, identifying regions of disproportionately high anemia prevalence at a county level (Chapman University, 2025). This comprehensive information helps to the healthcare providers to set specific goals, quantify improvements, and adjust practices based on changes in parameters (Cassidy et al., 2022).

AI can integrate with diverse data sources-including electronic health records, genomic data, and even satellite imagery-to enhance analytical capabilities. Comprehensive integration of AI data improves the speed and accuracy of health surveillance, enables more responsive and relatable public health interventions (Paramasivan, 2023).

#### 4.4.2 Resource allocation and targeted interventions

AI application can assist to standardize treatment recommendations and providing equal opportunities in delivery of patient care without compromising human factors. Detecting the patterns of disparities among patient care and tracking differences in diagnoses and access rates (Wang and Bertrand, 2025). This allows public health programs to better allocate resources and more specifically target interventions to vulnerable groups, helping to fill existing health gaps (Miyoshi, 2025).

In the area of health literacy among diverse population AI can also help develop evidence—based communication that is tailored to the needs of the populations, through making public health services more accessible (Bharel et al., 2024). Health literacy evidence is important for delivering information on anemia prevention, in nutritional management and screening, to potentially hard-to- reach communities (Wang and Bertrand, 2025). The enhancement of the human capability in public health efforts increase with ability of AI to analyze and translate machine-and human-based inputs into models helps formulate potential options for information or action (Bharel et al., 2024). The overview of AI Applications in Anemia Management was given in Table 3.

**Table 3:** Overview of AI applications in anemia management.

Application area	Specific AI technologies/models	Key functionalities/interventions	Reported effectiveness/accuracy	Relevant snippet IDs
Early diagnosis and screening	AI-powered smartphone apps	Non-invasive Hb estimation from fingernail images	Mean absolute error ±0.7 g/dL (improving to ±0.50 g/dL for Hb > 10 g/dL); 1.4 M + tests, 200 K + users	38
	Deep Learning (VGG16, ResNet-50, InceptionV3)	Non-invasive anemia detection from conjunctiva images	AUC 0.97; SVM accuracy 78.90–85%	11
	ML Models (CNN, SVM, Naïve Bayes, XGBoost, Catboost, Random Forest, ELM)	Anemia detection and classification from blood tests/images	CNN 90.27%, Naïve Bayes 89.96%, XGBoost 100%, Catboost 97.6%, RF 95.49%, ELM 99.21%	11
Personalized nutrition	ML/DL algorithms, IoT-based systems	Tailored dietary recommendations based on health data, genetics, wearables	Improved glycemic control, metabolic health, psychological well-being; statistically significant improvements in AI groups	26
Clinical management systems	Anemia Control Model (ACM) (Artificial Neural Network)	Personalized ESA and iron dosing for hemodialysis patients	Decreased darbepoetin consumption, increased on-target Hb (70.6 to 76.6%), reduced Hb fluctuation, reduced hospitalization risk	40

## 5 Challenges and ethical considerations in AI–based anemia interventions

There are various ethical consideration challenges despite the promising advances in the integration of AI into anemia interventions particularly among young adult women who faces significant challenges and raises critical ethical considerations. The challenges and Ethical Considerations in AI for women’s Health were given in Table 4.

**Table 4:** Challenges and ethical considerations in AI for women’s health.

Category of challenge	Specific issues	Impact on women’s health/anemia	Proposed mitigation strategies
Algorithmic bias	Male-centric training data; Underrepresentation of women in clinical trials; Lack of diversity in AI development teams	Misdiagnosis/under diagnosis; Exacerbation of health disparities for women and marginalized groups; Reinforcement of existing inequities	Inclusive data collection; Continuous bias monitoring and auditing; Multidisciplinary development teams; Involvement of underrepresented populations
Data privacy and security	Unauthorized access; Data breaches; Misuse of sensitive data (e.g., reproductive health); Cloud security vulnerabilities; Lack of transparency in data sharing	Erosion of trust; Risk of personal harm (e.g., for reproductive health data); Hindered adoption due to privacy fears	Data anonymization; Minimizing data collection; Strong encryption; Clear consent processes; Robust, unified regulatory frameworks
Accessibility and digital literacy	High implementation costs; Limited compatibility with existing infrastructure; Digital divide (unequal access to technology); Lack of digital literacy	Unequal access to AI benefits; Exacerbation of health disparities for vulnerable populations (e.g., low-income, rural)	Affordable digital health technologies; Widespread digital literacy programs; Addressing socioeconomic barriers to technology access
Transparency and trust	“Black-box” algorithms (lack of explainability); Patient fears of errors/malfunctions; Concerns about data privacy/sharing	Erosion of trust in AI systems and healthcare providers; Resistance to adoption; Difficulty for clinicians to interpret AI decisions	Open-source AI models; Clear communication about AI use; Collaborative oversight (policymakers, providers, developers); Patient-centered policies
Regulatory gaps	Rapid tech development outpacing regulations; Global fragmentation and inconsistent laws; Lack of clear accountability	Unclear safety and efficacy standards; Gaps in compliance and oversight; Potential for harm without recourse; Hindered responsible innovation	Unified global frameworks; Industry-led standards; Clear definition of roles and responsibilities; Proactive regulatory adaptation

### 5.1 Algorithmic bias and health disparities in women

AI systems, despite of their perceived objectivity they are inherently reflections of the data they are trained on and the biases embedded in their design processes (Most, 2025). Historically, a significant biomedical research and clinical trials have been male—centric by leading on the other hand an under representation of women in the research datasets (Most, 2025; Karpel et al., 2025). The data imbalance results in AI models that may misdiagnose, underdiagnose, or be significantly less accurate when applied to female patients as diseases can present differently in women compared to men (Most, 2025). The lack of diversity within the AI development teams further contributes to blind spots and limits the perspectives informing model design and evaluation (Most, 2025). Even generative AI is trained on vast online content that can perpetuate and amplify cultural stereotypes (Most, 2025).

Differences in the inherent biases in historical medical data, particularly the male—centric focus and women’s under—representation, constitute a significant threat to egalitarian AI application; without purposeful and continual efforts to diversify training data and development teams, AI runs the risk of not merely reproducing but actively amplifying existing health inequities for young adult women, limiting its potential for beneficial effect. This algorithmic bias can increase existing health disparities, particularly among marginalized populations such as Black, Indigenous, and other communities of colour women, LGBTQ+ communities, and people with disabilities (Accuray, 2025).

To decrease algorithmic bias for mitigation strategies include implementing comprehensive data collection practices to ensure diverse demographic representation, ongoing monitoring and review of AI results to identify and remove bias early, supporting multidisciplinary development teams, and actively involving representatives of disadvantaged populations in the design and evaluation process (Accuray, 2025).

#### 5.1.1 Reducing bias requires a multifaceted approach

*Comprehensive data collection*: ensure training datasets are diverse and representative of the entire target population, covering different genders, races, ethnicities, and socioeconomic backgrounds (Hattab et al., 2025).

*Bias detection and mitigation*: to identify and address biases promptly conducting rigorous bias testing using reduction algorithms and continuous monitoring of AI outcomes are necessary (Hattab et al., 2025).

*Transparency and accountability*: promote explainable AI to help healthcare professionals understand AI decision—making, build trust, and support informed human oversight (Donins and Behmane, 2023). Transparency is essential for building trust, especially in healthcare, where accountability for errors is critical (Yu et al., 2024).

### 5.2 Data privacy and security concerns in health apps

Patient data protection is a major concern for AI technologies in healthcare, as these technologies rely on large amounts of medical data (Khalifa and Albadawy, 2024). Misuse of data (especially when transferred between institutions without sufficient oversight), significant risks include unauthorized access, data breaches, and vulnerabilities associated with cloud—based AI application (Alation, 2025). Concerns are particularly heightened for women’s reproductive health apps, where sensitive user information is subject to evolving governmental regulations and varying state—lavel laws, posing risks of privacy breaches and misuse (Zadushlivy et al., 2025). Lack of user transparency and protection regarding how data is stored and shared with third parties, even for marketing or analytical purposes (Zadushlivy et al., 2025). Consumer surveys consistently reveal high levels of concern about online privacy with a majority are believing AI poses a significant to their personal information (Puntoni et al., 2021).

The extensive collection of sensitive health data by AI applications, combined with a persistent lack of transparency regarding data usage and evolving, fragmented regulatory landscapes, creates significant privacy risks that can influence user trust and hinder widespread adoption (Williamson and Prybutok, 2024). This is mainly relevant for young women who share highly personal and sensitive information. For mitigation strategies minimizing data collection, collecting only what is essential, establishing clear and comprehensive consent processes: and developing strong, unified regulatory frameworks will control this (Alation, 2025). This implies that technological advancement in AI is not accompanied by commensurate ethical safeguards and it required clear, adaptable regulatory frameworks, for public resistance and ultimately full potential in improving health outcomes.

### 5.3 Accessibility, digital literacy, and socioeconomic influences on adoption

Despite AI’s potential to improve access to healthcare, its actual uptake in healthcare has been slower than anticipated, partly due to high implementation costs and limited compatibility with existing hospital infrastructures (Shah, 2024). Pre-existing socioeconomic disparities, including limited digital literacy and unequal access to technology, create a new form of “digital divide” that could prevent vulnerable young women, who often bear the highest burden of anemia, form fully benefiting from this innovation (Alarsaheb, 2025; Al-Khassawneh, 2023).

Socioeconomic factors, like income levels and educational attainment will significantly influence both access and the effective adoption of digital health technologies (Davidson et al., 2022). The “digital divide” remains a critical barrier; ensuring affordable digital health technologies and widespread digital literacy programs are crucial for equitable access (de Graaf et al., 2025). Initiatives of digital literacy are identified as promising interventions to empower women to engage critically with AI technologies, understand their implications, and help mitigate gender disparities in the digital health space (Karpel et al., 2025). Without these primary efforts, AI could inadvertently exacerbate existing health inequalities by primarily benefiting those who already possess the means to access and effectively utilize the technology, thus failing to impact the most vulnerable.

### 5.4 Transparency, trust, and regulatory gaps

Many AI algorithms operate as “black boxes” making it difficult for users to understand, how decisions or recommendations are generated including healthcare professionals and patients (Alation, 2025). This opacity inherently undermines trust. Patient in AI in healthcare is further hindered by fears of diagnostic errors or device malfunctions, the lack of transparency in AI decision—making, and significant concerns about data privacy and unauthorized sharing with third parties (Alation, 2025).

Establishment of comprehensive regulatory frameworks which leads to global fragmentation and inconsistent laws that create in compliance and oversight of AI technology (Alation, 2025). The “black-box “nature of many AI algorithms and the lagging, fragmented regulatory frameworks critical governance gap threatening patient trust and hindering ethical, widespread adoption (Lin, 2024). This regulatory vacuum implies a lack of clear accountability and standardized safety and efficacy measures. There is an urgent need for collaborative oversight involving policymakers, healthcare professionals, and tech developers to ensure responsible innovation (Alation, 2025). Accountability for the performance and outcomes of AI algorithms must be clearly defined and enforced (Vij, 2024). Without robust, proactive governance including clear consent processes, bias mitigation, and defined responsibilities are AI’s potential benefits will be undermined by legitimate concerns about safety, fairness, and accountability, particularly when dealing with sensitive health data and vulnerable population like young women (Emma, 2024).

### 5.5 Integration with existing healthcare systems and workflows

Significant logistical challenges in AI is follows as healthcare IT infrastructure, clinical workflows, and administrative processes also presents (Wright and Fairley, 2025). Like Seamless interoperability with electronic health records (EHRs), imaging equipment, and other healthcare technologies are essential to avoid disruptions and inefficiencies (Donins and Behmane, 2023). The lack of validation methods also limits the AI’s acceptance in medical practice (Wright and Fairley, 2025).

Interdisciplinary collaboration is required for successful integration among clinical, IT, and AI teams (Wright and Fairley, 2025). By using interoperability standards and open APIs can facilitate compactivity (Hattab et al., 2025). Hence, the rapid pace of technological advancements often outpaces the development of regulates various frameworks and practical integration strategies by creating implementation gaps (Alation, 2025).

### 5.6 Ethical and regulatory frameworks

Ethical implications are involved in AI in healthcare extended beyond the data privacy and bias to encompass issues of accountability, informed consent and the nature of human-AI collaboration (Gifari et al., 2021).

#### 5.6.1 Accountability and liability

AI- driven systems take critical decisions in treatment or generate errors, determining who is responsible becomes complex (Donins and Behmane, 2023). Placing full responsibility on clinicians for AI-driven errors may be unreasonable and holding the machine responsible is illogical (Akhtar, 2024). This shift necessitates toward shared responsibility among healthcare institutions and involved parties, Clinicians and AI technologists combinly has to investigate on the impact of medical litigation (Khalifa and Albadawy, 2024). The opacity of “black box” AI systems further complicates accountability, as their decision-making processes are not easily decipherable (Alation, 2025).

#### 5.6.2 Informed consent and patient autonomy

Patients should be properly informed about the role of AI in their diagnosis and treatment, and they should be free to consent or opt out if they are uncomfortable. Automated decision—making with a major impact on individuals should ideally include human review or an opt-out option (WHO, 2025). It protects the patient liberty and fosters trust in the healthcare system (Alation, 2025).

### 5.7 Over–reliance and human–AI collaboration

Even though AI technology supports healthcare services, excessive dependence on AI technology may devalue human judgment and cause errors to be overlooked with potentially fatal consequences (Bouderhem, 2024). Untrustworthy predictions with AI systems, especially large language models, can generate “hallucinations” (Yu et al., 2024).

With a collaborative approach AI integration should utilize AI as a supplement to human to human—led practices rather than a replacement (Bharel et al., 2024). Human-in—the -loop processes are a vital step toward increased accuracy and trust in AI—where technology is constantly refined by human oversight. This cycle of trustworthiness contributes to improved system reliability, reduction of errors, and fostering of trust which in term fosters AI transparency and acceptance among healthcare workers (Hattab et al., 2025).

The main goal is a balance between the complexity of AI algorithms and the need for transparency by ensuring that AI tools positively on doctor and patient relationships by fostering empathy, shared decision—making and trust (Nasir et al., 2025).

## 6 Future directions and unmet needs

Integrating arterial intelligence into the treatment of anemia in young women is promising but there is still significant potential for development and unmet needs. Future efforts should be directed toward improving AI capabilities, overcoming existing limits, and providing fair access to these transformative technologies (Alanezi, 2024).

### 6.1 Advancing AI capabilities for anemia

Future research and development on arterial interleave to combat anemia should on several key areas as follows.

#### 6.1.1 Enhanced diagnostic precision

An AI model shows high accuracy in detection of anemia and there is a need for refinement and improvement of diagnosis, especially in differentiating of various types of anemia (e.g, iron deficiency thalassemia, and B12 deficiency) that may present with similar symptoms (Saputra et al., 2023). Developing AI models can integrate features from blood smear images with clinical data from CBC tests, even with limited patient samples has shown promise for improving diagnostic accuracy. The goal is to create more robust and reliable models for clinically relevant decision—support systems (Sehar et al., 2025).

#### 6.1.2 Personalized intervention and predictive analytics

The management of anemia still largely ignores AI’s prominent use for personalized medicine. Future research should concentrate on using AI to examine each person’s distinct genetic compositions, lifestyle and environmental influences to create highly focused and successful interventions (Bhatia et al., 2024). Using real-time biometric data, this involves optimizing treatment regimens and personalized nutrition programs based on gut microbiota composition, biomarkers, and prediction models that predict individual reactions to iron supplements or other therapies (WHO, 2025). Improved accuracy in predicting the course of diseases and identifying high risk individuals through proactive and preventative therapy will be made possible (Mohamed et al., 2024).

#### 6.1.3 Integration of multi–modal data

The future of AI in anemia management lies in its ability to integrate wider array of multi—modal data including imaging, genetic, clinical, and environmental data (Xu et al., 2024). Data integration enables the most comprehensive and accurate access to a wide range of patient cares, enabling AI to identify complex interactions and subtle patterns that influence anemia risk and response to treatment (Morozov et al., 2021).

### 6.2 Addressing unmet needs women‘s health and ANEMIA

AI can help solve many important problems in women’s health, especially in the context of anemia.

#### 6.2.1 Bridging diagnostic and treatment gaps

Many women suffer from various conditions, including menorrhagia, gynecological conditions, which often go undiagnosed or underdiagnosed (Pasricha et al., 2021). Artificial intelligence can aid in the early diagnosis of diseases, predict health risks, and provide proactive health information (García-Micó and Laukyte, 2023). For hemoglobin assessment common laboratory tests are expensive, time-consuming, and require extensive clinical infrastructure. Whereas using non- invasive screening methods, such as mobile phone apps, significantly increasing accessibility to testing, especially in remote and underdeveloped areas (Dasl and Gupta, 2024).

#### 6.2.2 Enhancing accessibility and equity

Despite AI’s potential there is a prevailing concern that the latest innovations tend to be applied first in higher-resourced settings lead to potentially exacerbating existing health disparities (Yu and Zhai, 2024). A key need is to make sure that AI solutions for anemia are designed and used with a clear focus on fairness, inclusivity, and real impact for different population. This includes non-English speakers and people from low-income backgrounds. It means creating tools that function well in low- bandwidth settings and address language barriers through AI-powered natural language processing (WHO, 2025). It is important that AI can help all patients, not just those who are digitally connected to close healthcare gaps (Wang and Bertrand, 2025).

#### 6.2.3 Continuous validation and regulatory oversight

For AI nutrition recommendations and tools for managing anemia to be widely accepted in clinical practice, ongoing validation through real-world clinical trials is necessary (Kassem et al., 2025). Required standardized validation methods to make sure AI—assisted dietary assessment tools and other interventions which are clinically accurate and reliable. Policymakers, medical experts, and technology developers must work together to ensure ethical implementation of AI systems and build trust (Wright and Fairley, 2025).

## 7 Conclusion

In young adult women, anemia remains a terrible public health challenge, particularly in low- and middle-income countries with persistently high prevalence rates despite ongoing international efforts. Anemia is caused by a complex interplay of nutritional deficiencies, profound socioeconomic disadvantages, and unique physiological and genetic vulnerabilities. Perpetuating successive generations of poverty and diminished human potential, the physical social, and economic impacts are vast. The inadequate efficacy of various existing therapies underscores the urgent need for creative and scalable solutions.

Artificial intelligence offers a transformative opportunity to tackle these challenges. AI-powered, on- invasive diagnostic tools—such as smartphone—based nail analysis and conjunctival imaging-provide accurate, low-cost and accessible screening methods that can overcome barriers in traditional healthcare infrastructure, thereby democratizing early detection in underserved populations. Additionally, advanced machine learning models not only improve diagnostic accuracy but can also classify specific types of anemia, crucial for guiding targeted treatments and optimizing patient care. Beyond diagnosis, AI- Driven personalized nutrition systems deliver precise, adaptive recommendations tailored to an individual’s unique biology and lifestyle, moving well beyond one-size-fits-all dietary advice to effectively combat nutritional anemia. Early clinical case studies also indicate that AI-driven management systems can lead to tangible improvements in patient outcomes, including better hemoglobin control, reduced medication use, and decreased hospitalization rates, especially for chronic diseases.

However, the effective and equitable integration of AI into anemia interventions faces significant challenges. Algorithmic biases-often rooted in historically male-biased data and a lack of diversity within development teams- pose serious risks of replicating and even worsening existing health disparities for women. Concerns over data privacy and security, compounded by opaque data usage practices and fragmented regulatory environments, can undermine user trust and hinder widespread adoption, especially when dealing with sensitive health information. Moreover, socio- economic inequalities and the digital divide threaten to limit access to AI innovations for the most vulnerable populations. The “black box” nature of many AI algorithms, alongside unclear regulatory frameworks, creates a governance gap that demands clear, collaborative, and tailored ethical and legal guidance.

For AI to fully realize its potential in promoting health equity in anemia treatment for young women, a multifaceted approach is essential. This includes proactive efforts to minimize algorithmic bias through diverse, representative data collection and inclusive development teams, robust data protection and transparent consent processes and global initiatives aimed at bridging the digital divide via comprehensive digital and programmatic literacy programs. Furthermore, establishing clear, consistent, and adaptable regulatory frameworks ai critical to building trust and ensuring the responsible, ethical, and effective integration of AI into health systems worldwide.

The future of anemia management for young adult women will undoubtedly be shaped by the thoughtful application of AI. By advancing capabilities for enhanced diagnostic precision, personalized interventions, and multimodal data integration—while consciously addressing accessibility, equity, and ethical oversight—AI can play a pivotal role in achieving meaningful and sustainable reductions in the global anemia burden.
